# A Triad of Trichobezoar: Rapunzel Syndrome, Severe Malnutrition, and Cerebral Venous Thrombosis

**DOI:** 10.7759/cureus.38016

**Published:** 2023-04-23

**Authors:** Minal Shastri, Darshankumar M Raval, Vaishnavi M Rathod, Shashwat Mallik, Shahin Khan

**Affiliations:** 1 Internal Medicine, Government Medical College, Baroda, Vadodara, IND

**Keywords:** trichobezoar, trichotillomania, rapunzel syndrome, malnutrition, cerebral venous thrombosis

## Abstract

Trichobezoar is a rare condition almost exclusively seen in young females presenting with non-specific abdominal complaints and a history of psychiatric illness. In most patients, it is confined within the stomach; however, in some severe cases, it extends through the pylorus into the duodenum, jejunum, ileum, or even colon, known as Rapunzel syndrome. Conventional treatment includes laparotomy and psychiatric counseling to prevent relapses. We report the case of an 18-year-old female with no previous history of medical or psychiatric illness who presented with chief complaints of upper abdominal pain, nausea, occasional vomiting for the last six months, and generalized edema for the last three days. On examination, pallor, anasarca, and a palpable abdominal lump were present. On blood investigations, severe malnutrition was seen in the form of severe iron deficiency anemia and severe protein deficiency. Radiological evaluation revealed a large trichobezoar on the CT abdomen and endoscopy, whereas CT venography of the brain, done for persistent headache, showed hyperdense thrombi in the cortical veins. Exploratory laparotomy was done to remove trichobezoar, followed by medical management of malnutrition, cerebral venous thrombosis (CVT) with anticoagulants, and psychiatric counseling for trichobezoar. The association between trichobezoar, malnutrition, and CVT in our case is a further area of research.

## Introduction

The proximal gut obstructed by a ball of hair is a rare condition known as trichobezoar, almost exclusively affecting young females between 15 and 20 years of age with a history of trichotillomania and trichophagia [1]. In most cases, the trichobezoar is confined within the stomach; however, in some severe cases, the trichobezoar extends through the pylorus into the duodenum, jejunum, ileum, or even colon, which is called the Rapunzel syndrome [2].

The accumulated hair within the stomach and intestine is resistant to digestion, frequently leading to a spectrum of nonspecific clinical findings, including early satiety, anorexia, vomiting, and vague abdominal pain, and occasionally presents as trichobezoar-induced bowel or gastric outlet obstruction. Traditional therapy has included endoscopy, with a subsequent combination of laparoscopy and/or laparotomy, and associated psychiatric intervention [3]. Here, we have reported a rare case of a large trichobezoar leading to severe malnutrition and its complication, cerebral venous thrombosis (CVT), in an 18-year-old female.

## Case presentation

An 18-year-old, unmarried female presented to the medicine outpatient department with chief complaints of dull aching type of upper abdominal pain, nausea, occasional vomiting, intermittent constipation, weight loss, and easy fatigability for the last six months, which were aggravated in the last three days. In addition, the patient also had complaints of breathlessness on exertion (NYHA Grade II), paroxysmal nocturnal dyspnea, persistent dull aching type of headache, and amenorrhea for the last two months. The patient had developed generalized swelling all over the body (anasarca), starting from the lower limbs and extending to the entire body within three days. There were no complaints of diarrhea, fever, cough, cold, chest pain, palpitation, joint pain, oral ulcers, hair loss, decreased urine output, burning micturition, hematuria, bleeding from any site, vaginal discharge, or difficulty in vision. The past history of previous medical and psychiatric illnesses was not significant.

On general examination, the patient was vitally stable, except for pallor, anasarca (pitting type, more marked in lower limbs), and koilonychia. On systemic examination, per abdomen examination revealed a palpable lump in the left hypochondriac and epigastric regions. On examination of the cardiovascular, respiratory, and central nervous systems, no abnormality was detected. The routine investigations were suggestive of severe anemia with mild thrombocytosis and severe protein deficiency, indicating severe malnutrition (Table 1).

**Table 1:** Serial routine investigations g/dL, gram per deciliter; mg/dl, milligram per deciliter; mEq/L, milliequivalents per liter

Investigations	On admission	Day five	On discharge
Hemoglobin (g/dl)	4.5	8.4	11.1
Total count (per cumm)	7000	8600	11300
Neutrophil count (%)	54	69	84
Lymphocyte count (%)	44	29	14
Eosinophil count (%)	1	1	1
Monocyte count (%)	1	1	1
Platelet count (per cumm)	5.56 lakhs	5.37 lakhs	2.39 lakhs
Urea (mg/dl)	60	18	25
Creatinine (mg/dl)	0.61	0.60	0.71
Total bilirubin (mg/dl)	0.5	0.6	0.7
Direct bilirubin (mg/dl)	0.2	0.2	0.3
Indirect bilirubin (mg/dl)	0.3	0.4	0.4
S. sodium (mEq/L)	135	131	134
S. potassium (mEq/L)	4.6	3.5	3.8
S. total protein (g/dl)	3.9	4.3	5.9
S. albumin (g/dl)	1.6	2.0	3.0

This was confirmed by further investigations such as RBC indices, peripheral smear, and iron study, suggestive of severe iron deficiency (Table 2).

**Table 2:** Other investigations PCV, packed cell volume; MCV, mean corpuscular volume; MCH, mean corpuscular hemoglobin; MCHC, mean corpuscular hemoglobin concentration; RDW, red cell distribution width; fL, femtoliter; pg, picogram; g/dl, gram per deciliter; ng/dl, nanogram per deciliter; INR, international normalized ratio; aPTT, activated partial thromboplastin time; RBS, random blood sugar; LDH, lactate dehydrogenase; SGPT, serum glutamic pyruvic transaminase; SGOT, serum glutamic oxalacetic transaminase; ALP, alkaline phosphatase; TSH, thyroid stimulating hormone; U/L, units per liter; HIV, human immunodeficiency virus; HBsAg, hepatitis B surface antigen; HCV, hepatitis C virus

Investigations	Result
PCV (%)	20.8
		MCV (fL)	66.2
				MCH (pg/cell)	18.5
						MCHC (g/dl)	27.9
								RDW (%)	18
										S. ferritin (ng/dl)	8.34
S. iron (microgram/dl)	68								
TIBC (microgram/dl)	263								
Saturation %	25.86								
Erythrocyte sedimentation rate (millimeter/hour)	16								
Reticulocyte count (%)	2
Peripheral smear	Moderate microcytic hypochromic RBCs, mild anisopoikilocytosis, mild thrombocytosis
Malarial parasite	Negative
Prothrombin time test (seconds)	14.0
Prothrombin time control (seconds)	13.7
INR	1.02
aPTT test (seconds)	33.9
aPTT control (seconds)	33.8
Urine routine and microbiology	Normal
RBS (mg/dl)	97
LDH (U/L)	691
SGPT (U/L)	52
SGOT (U/L)	118
ALP (U/L)	100
Serum TSH (IU/L)	4.821
Direct and indirect Coombs test	Negative
HIV	Negative
HBsAg	Negative
HCV	Negative
Blood group	AB+

Mild elevation in liver transaminases was suggestive of possible involvement of the liver as well. The thyroid function test, urine examination, blood sugar level, and coagulation profile were within normal range. The viral markers screening and direct and indirect Coombs test were negative (Table 2).

**Figure 1:**
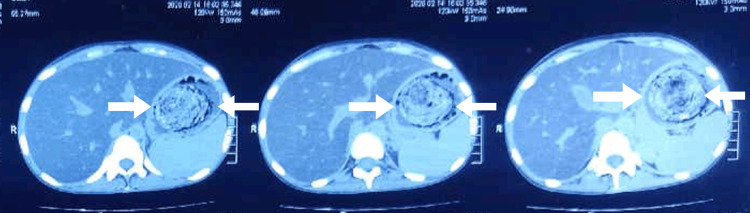
Axial section of CT abdomen and pelvis showing trichobezoar CT, computed tomography

**Figure 2:**
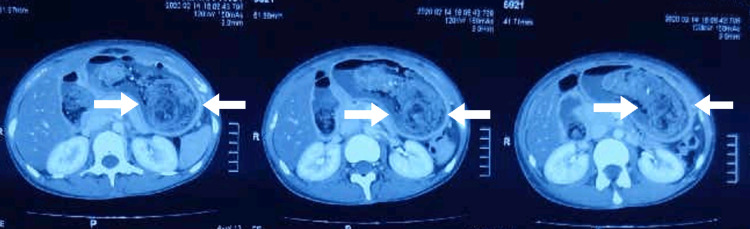
Axial section of CECT abdomen and pelvis showing trichobezoar CECT, contrast-enhanced computed tomography

**Figure 3:**
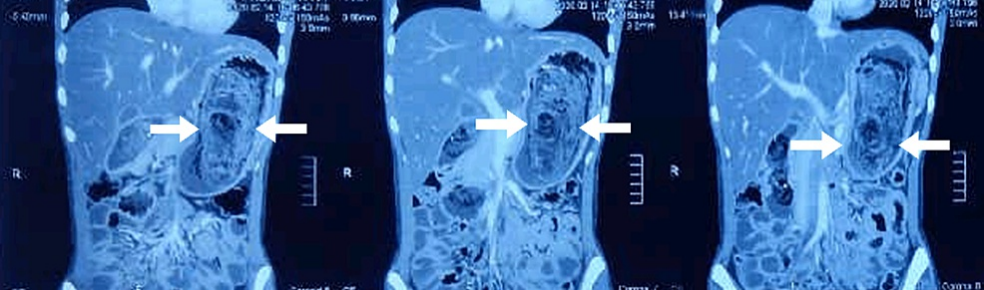
Coronal section of CECT abdomen and pelvis showing trichobezoar CECT, contrast-enhanced computed tomography

**Figure 4:**
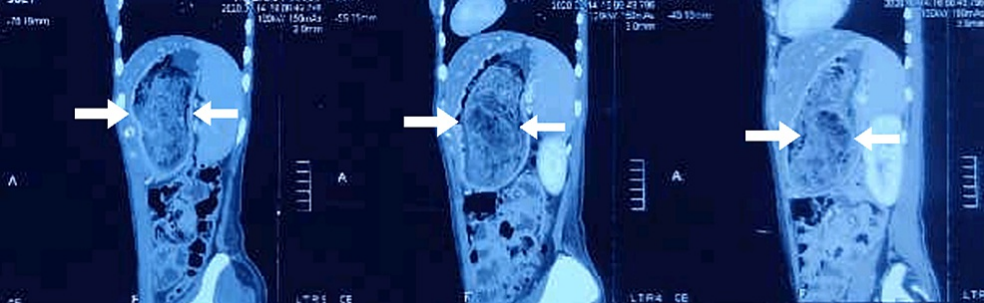
Sagittal section of CECT abdomen and pelvis showing trichobezoar CECT, contrast-enhanced computed tomography

Ultrasonography of the abdomen and pelvis was done for the evaluation of the lump, which did not show any abnormality except for the moderate fluid in the pelvic space (possibly due to severe protein deficiency). Therefore, further evaluation was carried out by CT abdomen (plain and contrast), which revealed a bezoar-like mass extending from the stomach to the duodenojejunal junction and hepatomegaly with severe fatty changes (Table 3, Figures 1-4).

**Table 3:** Radiological investigations CT, computed tomography

Investigations	Remarks
Ultrasonography of the abdomen and pelvis	Moderate free fluid in the pelvis, otherwise normal.
CT abdomen (plain and contrast)	Mixed density large oblong lesion (with scattered calcification as well) giving a mottled appearance occupying the entire stomach and distending it as well as extending into the pylorus and up to the duodenojejunal junction, likely to represent bezoar. Hepatomegaly with severe fatty changes and mild splenomegaly.
CT head plain	Suspicious hyperdensity in the region of the vein of Galen and straight sinus, maybe thrombus. Diffuse cerebrocortical atrophy.
CT venography of the brain	Hyperdense thrombi in the straight sinus, the vein of Galen, and bilateral distal internal cerebral veins causing total occlusion.
Esophago-gastro-duodenoscopy	A large trichobezoar occupies the whole stomach and extends through the pyloric channel seen in the stomach. Ulcerated pyloric margin present. Not possible to enter into the duodenum. Normal esophagus.

**Figure 5:**
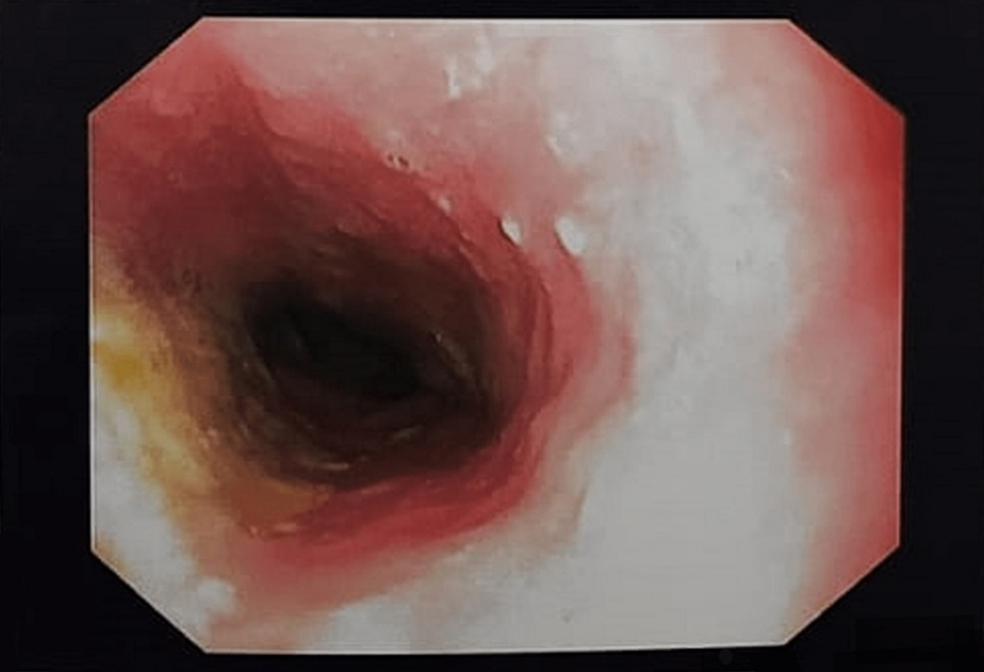
Endoscopy showing a normal esophagus

**Figure 6:**
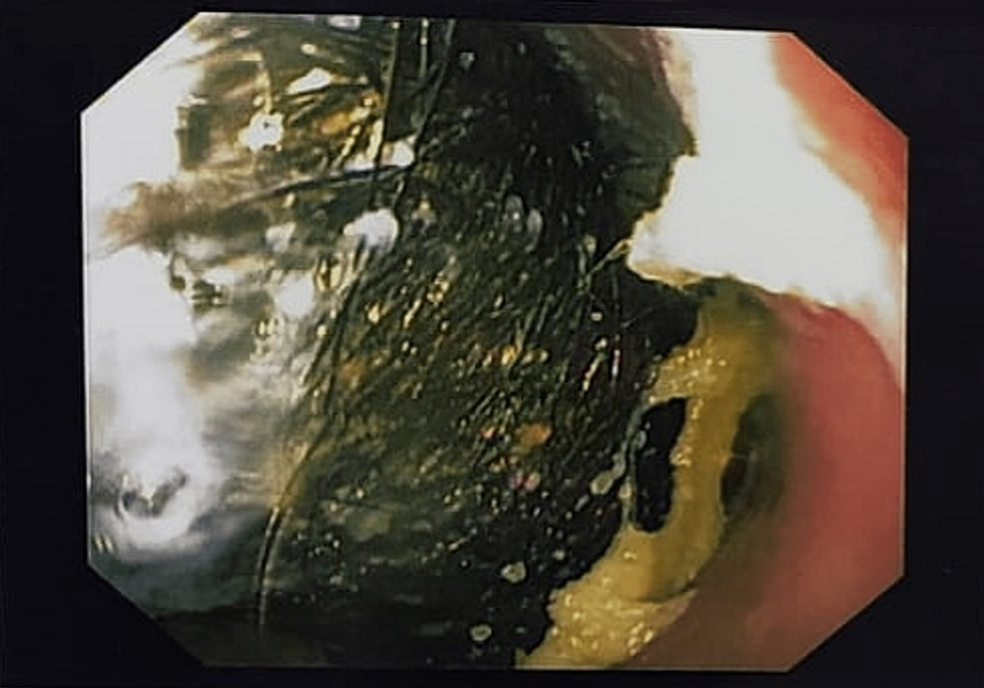
Endoscopy showing trichobezoar in the stomach

**Figure 7:**
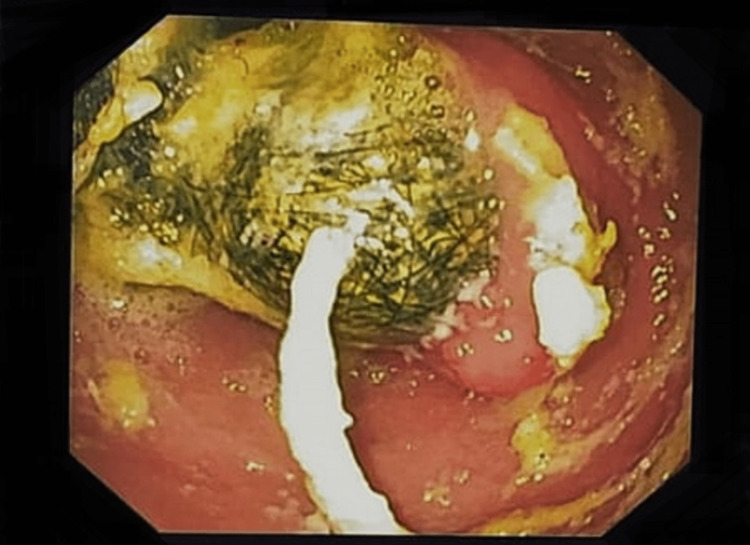
Endoscopy showing trichobezoar extending through the pyloric channel

Esophago-gastro-duodenoscopy confirmed the diagnosis of trichobezoar (Figures 5-7, Video 1).

 

**Video 1:** Esophago-gastro-duodenoscopy

**Figure 8:**
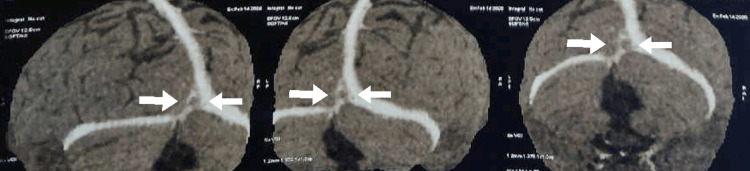
CT venography showing straight sinus and lateral sinuses CT, computed tomography

**Figure 9:**
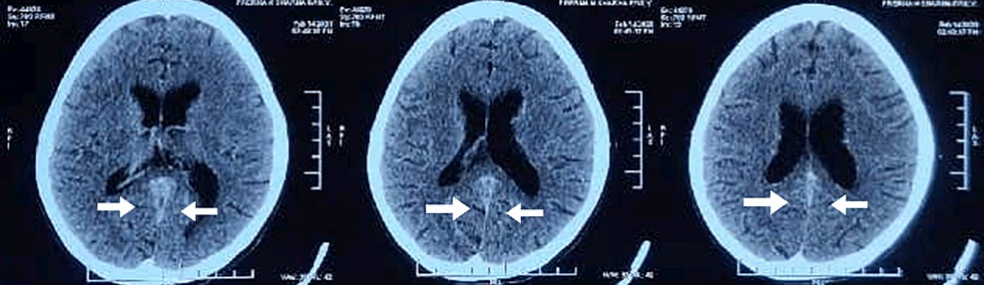
Axial section of CT venography showing thrombus of the straight sinus CT, computed tomography

The patient was simultaneously evaluated for amenorrhea and persistent headache. Gynecological consultation attributed amenorrhea to psychiatric causes and severe malnutrition. Ophthalmologic examination including fundus and vision checkup was normal without papilledema. The psychiatry consultation revealed a history of pulling hair due to itching on the scalp for the last year; however, no other psychiatric disorder was found. She denied any history of eating hair. For the evaluation of persistent headache, a CT head (plain) was done, which showed the possibility of thrombus in the vein of Galen and straight sinus regions, further confirmed by CT venography of the Brain, which is suggestive of CVT (Table 3, Figures 8-9).

**Figure 10:**
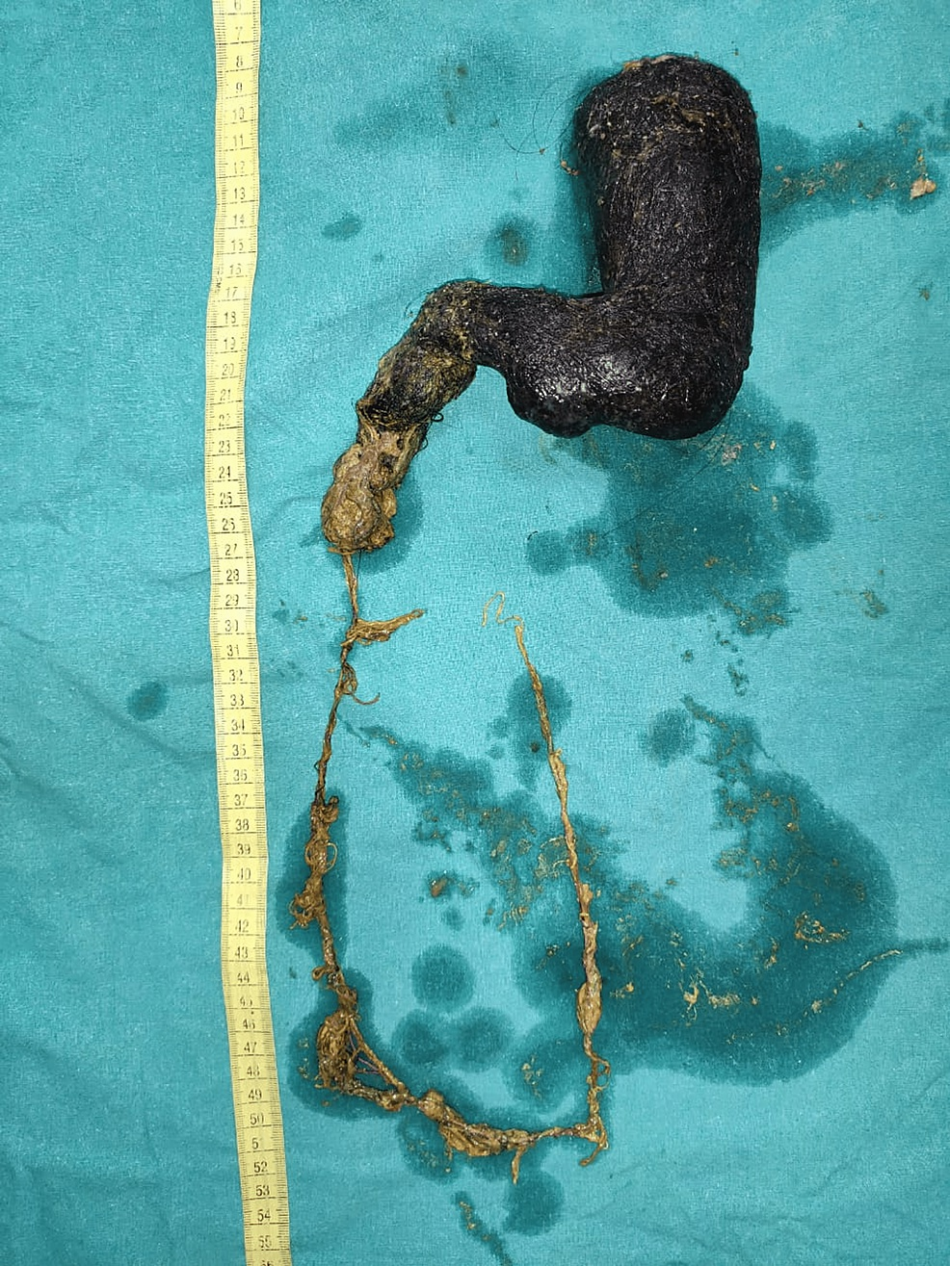
A large trichobezoar extending from the stomach to the proximal jejunum removed by exploratory laparotomy

Once the diagnosis of trichobezoar was made, surgical advice was sought. After improving her general pre-operative condition by transfusing packed red cells, a large trichobezoar extending from the stomach to the proximal jejunum was removed by exploratory laparotomy (Figure 10).

The patient was referred back to the medicine department for further management of severe malnutrition and CVT and was closely monitored by both medicine and surgical departments in the postoperative period for any bleeding complications following anticoagulant therapy. After 10 days of hospital stay, she improved clinically and symptomatically. She was finally discharged on an oral anticoagulant, with advice to follow up regularly for the management of CVT.

## Discussion

Trichobezoar is an unusual condition and is usually found in young psychiatric females, who often deny eating their own hair. It is caused by the pathological ingestion of hair, which remains undigested in the stomach and sometimes may extend into the small intestine, the condition known as Rapunzel syndrome [4]. Bezoars may present with abdominal pain, nausea/vomiting, early satiety, weight loss, intestinal obstruction, and ulceration leading to bleeding and/or perforation. Rarely intussusception can also occur [5].

Many cases of trichobezoar have been reported; however, our case has several unusual findings. Our patient was a young female who had a history of pulling hair from her scalp due to itching, but no major psychiatric illness was found in her psychiatric evaluation. Moreover, she presented with severe malnutrition and its complications, such as severe iron deficiency anemia, severe protein deficiency, generalized edema (anasarca), amenorrhea, hepatomegaly with severe fatty changes, and CVT. The occurrence of CVT in our patient could be attributed to severe malnutrition, protein deficiency, and severe fatty changes in the liver. However, further investigations are needed to establish a strong association between these conditions. Liver cell damage due to malnutrition sometimes leads to thrombotic conditions, as levels of anticoagulant proteins decrease [6,7].

As a diagnostic modality, endoscopy can differentiate trichobezoar from other types of bezoars and foreign bodies. However, the treatment of choice in patients with trichobezoar and Rapunzel syndrome should remain conventional laparotomy. The literature provides no evidence of the superiority of endoscopy or laparoscopy in terms of efficacy. The lack of invasiveness of these techniques does not seem to outweigh the inferior efficacy and complexity of these procedures [1]. Psychiatric consultation and follow-up are imperative along with acute surgical treatment to prevent relapses [1]. Our patient also underwent exploratory laparotomy and was then referred back to the medicine department for the management of severe malnutrition and CVT. As she was not suffering from any psychiatric illness, she was only counseled by the psychiatrist and was discharged after 10 days of hospitalization on an oral anticoagulant for CVT with advice to follow up for the management of CVT. Larger observational studies are required to investigate the possible relationship between malnutrition and thromboembolic conditions.

## Conclusions

Trichobezoar is a rare condition mainly seen in young females, and its severe form called Rapunzel syndrome is even rarer. Here, we have reported a patient with trichobezoar, who was later found to have Rapunzel syndrome, presented with severe malnutrition and its complications, one of them being CVT. However, whether CVT in our patient was an incidental finding or its causal association with malnutrition is a further area of research. After exploratory laparotomy for trichobezoar, our patient was primarily managed by medical interventions for malnutrition and CVT.
